# Experiences of auxiliary nursing trainees of poverty alleviation programme regarding nursing and nursing profession in Vhembe District, Limpopo Province

**DOI:** 10.4102/curationis.v42i1.1792

**Published:** 2019-08-06

**Authors:** Maria S. Maputle, Florance N. Baloyi, Livhuwani H. Nemathaga

**Affiliations:** 1 Department of Advanced Nursing Science, University of Venda, Thohoyandou, South Africa

**Keywords:** auxiliary nurses, employability, nursing profession, poverty alleviation strategy, orphans

## Abstract

**Background:**

The Limpopo Department of Health and Social Development introduced a system to train children from poor families as auxiliary nurses as a poverty alleviation strategy in 2003. The programme targeted the needy families, those who depended on social grants, orphaned or child-headed families. The programme has been implemented for more than 10 years and the experiences of trainees were never explored.

**Objectives:**

The purpose of this study was to explore the experiences of auxiliary nurse trainees of the poverty alleviation programme regarding nursing and the nursing profession.

**Methods:**

A qualitative approach was used. Purposive sampling was used to sample 15 auxiliary nurses who were trained under poverty alleviation programme in four of the seven hospitals of Vhembe District. Data were collected through in-depth individual interviews. One central question ‘As a beneficiary of poverty alleviation programme, could you describe your experiences of training as a nurse and of the nursing profession in this hospital’. Data were collected until no new information emerged. Note taking and a voice recording was performed to capture all the information reported by the participants. Open coding method was used to analyse data.

**Results:**

Two themes emerged, namely experiences of being a nurse and about nursing as a profession, and interpersonal relationships between auxiliary nurses and the ward staff.

**Conclusions:**

The Department of Health in Limpopo Province was commended and to be encouraged to develop other programmes as poverty alleviation strategy for other government departments, so that the affected children can have a wider career choice. The managers and supervisors in the wards to have moral and legal obligations to support initiatives that foster effective mentoring of neophyte nurses in the nursing profession.

## Introduction

Training and skills development available to vulnerable youth can be of help to upskill themselves and offers a better chance of escaping poverty (Mayombe 2014). Kgadima (2009) believed that South Africa is faced with the challenge of poverty and hunger. The move towards the realisation of the sustainable development goals (SDGs), goal (1) is: ‘End poverty in all its forms which is linked to the eradication of poverty and hunger by 2015, which in turn encourages developing countries to embark on poverty alleviation programmes’ (Sachs & McArthur 2005). In his South African National Congress statement, the ex-President of South Africa, Mr Thabo Mbeki, declared that 2003 was the year of the struggle against poverty and called upon all governmental departments to develop poverty alleviation programmes (ANC 2003). Hence, the Department of Health and Social Development in Limpopo Province under the then Member of the Executive Council (MEC) of Health, Honourable Mr Sekoati, initiated poverty alleviation programme.

The programme targeted orphans from poverty-stricken families to be trained as nursing auxiliaries. The government was aware that grants alone cannot sustain the lives of the communities. To address this, the discussion document on linking beneficiaries of social grants to economic opportunities was released by the government (Sekoati 2008). The document was based on the view that government just cannot provide grants and leave families and individuals vulnerable in other aspects of life, and that decent and sustainable work remains a cornerstone in the eradication of poverty (Sekoati 2008). Responding to this challenge, the MEC called upon communities in the Limpopo Province to utilise the opportunities created by the government. These included, amongst others, poverty alleviation programmes, one of which was auxiliary nursing training for individuals who passed Grade 12 and were from poverty-stricken families (Sekoati 2008).

A committee was appointed to identify and select trainees. The criteria used for selection of auxiliary nurses for training were: The applicant had to be from (1) a child-headed family and (2) a family where needy children received food parcels from the Department of Social and Welfare, was in possession of Grade 12 certificate and was aged below 40 years (Department of Health and Social Development 2003). According to the South African Nursing Council (SANC), candidates who are interested in nursing should have passed Grade 12. They can apply to the in-charge of a nursing education institution for admission to the course and be enrolled as an auxiliary nurse (SANC R2176 2005). Once the families were identified, candidates were interviewed and selected and they were then enrolled for training as auxiliary nurses in the seven hospitals of Vhembe District in Limpopo Province, namely Donald Frazer, Elim, Louis Trichardt, Malamulele, Musina, Siloam and Tshilidzini. The envisaged benefits of the programme to the department were filling in the vacancies, improving service delivery and reducing the burden for South African Social Security Agency and relatives. With regard to formulating a nursing care plan for a patient, auxiliary nurses offer noteworthy contributions by admitting patients and performing routine basic nursing procedures. According to the scope of practice in the *Nursing Act* 33 of 2015 of the auxiliary nurse (South African Nursing Council 2005), they must be supervised by a professional nurse when providing elementary nursing care to a health care user.

When the study was conducted, the programme had been implemented for more than 10 years where the orphans had been trained as auxiliary nurses through poverty alleviation programme. However, their experiences as auxiliary nurses had never been explored. Letshokgohla (2009) concurred that through this programme, the Department of Health and Social Development has trained 2000 orphans as auxiliary nurses from 2003 to 2015.

### Problem statement

The training of children from poor families and child-headed families as auxiliary nurses in hospitals of the Limpopo Province was initiated as a poverty alleviation programme. The Quality Assurance section at one hospital in Vhembe District indicated in the analysis of patient satisfaction questionnaires that 60% of the patients were not satisfied with the quality of care being provided by the auxiliary nurses. The report further indicated that auxiliary nurses did not respond to patients’ needs, especially those with disabilities, and they were not fed or changed if soiled. Hence, the purpose of this study was to determine the experiences of auxiliary nurses who trained under the poverty alleviation programme regarding the nursing and nursing profession in hospitals of Vhembe District in the Limpopo Province. The objective was to explore and describe their experiences of training as nurses and of the nursing profession.

### Research methods and design

A qualitative approach was used applying explorative, descriptive and contextual design. The population consisted of all auxiliary nurses who were trained under the poverty alleviation programme in hospitals of Vhembe District in the Limpopo Province. As documented in the Final Limpopo DHSD (2008/09-11 Annual performance Plan – Vote 7), for 2011, the target was to have 750 trained auxiliary nurses; however, it was not mentioned as to how many were trainees of the poverty alleviation programme.

Systematic random sampling was used to sample four of the seven hospitals. The list of hospitals was arranged in alphabetic order and the odd-numbered hospitals were included in the study, as follows: *Donald Frazer*, Elim, *Louis Trichardt*, Malamulele, *Musina*, Siloam and *Tshilidzini*. Purposive sampling was used to sample four auxiliary nurses from three hospitals and three from one hospital. In total, 15 auxiliary nurses who were trainees under the poverty alleviation programme in Vhembe District, Limpopo Province, were sampled. The sample size was determined by data saturation as postulated in De Vos et al. (2014).

#### Data collection

Data were collected between January and June 2015 through unstructured one-to-one interview method. One central question followed by probing was posed to the participants as follows: ‘As a beneficiary of poverty alleviation programme, could you describe your experiences of training as a nurse and of the nursing profession in this hospital’. Interviews were conducted by the student researcher under the supervision of two supervisors at the duty room during the lunch hour of participants. Data were collected in local language and verbatim statements were then translated into English by the language practitioner who is proficient in Tshivenda and Xitsonga. Interviews lasted for not more than 45 min. The probing questions were asked to encourage the participants to give additional information and for the researcher to get clarity on certain issues that arose during interview. Field notes were taken and a voice recorder was used to capture all the information reported by the participants.

#### Data analysis

The narrative data from interviews were analysed qualitatively through the open coding method as described in Creswell (2014). The method included these steps: all transcripts were read carefully by the researcher to get sense of the whole. A list of all similar topics was compiled. Data were then grouped according to categories and sub-categories. A literature control was performed to control the results of the study (Creswell 2014).

#### Measures to ensure trustworthiness

The criteria to ensure trustworthiness as outlined in Guba and Lincoln (1985) were used. Credibility was ensured by prolonged engagement which increased rapport and to clarify descriptions with participants through familiarity and through the use of the independent coder. The researcher collected data through field notes and in-depth individual interviews to ensure data triangulation. Member checking was performed to confirm and validate the findings with participants.

#### Ethical consideration

Ethical principles of confidentiality and anonymity were adhered to throughout the study. Ethical clearance (SHS/14/PH/05/1605) was obtained from the University Ethics Committee. Approval and permission to access the facility was obtained from the Limpopo Department of Health Research Committee and the Nursing Managers of the institutions. The participants signed the informed consent forms agreeing to participate in the study.

### Findings

The demographic profile of the participants is summarised in Table 1.

**TABLE 1:** Demographic profile of the participants.

Criterion	Characteristic	Frequency	Percentage
Age	23–25	6	40.0
	26–30	5	33.3
	31–35	2	13.3
	36–40	2	13.3
Gender	Women	10	66.6
	Men	5	33.3
Ethnicity	Black people	15	100.0
Level of education	Grade 12	15	100.0
Training as auxiliary nurse	1-year course	15	100.0
Previous experience	2–3 years	9	60.0
	4–6 years	6	40.0

The participants’ ages ranged from 23 to 40 years: five (33.3%) of the participants were men and 10 (66.6%) were women. All were black people, and they were in possession of a Grade 12 certificate to satisfy one of the criteria for selection of orphans to be trained as auxiliary nurses. All participants were trainees of the poverty alleviation programme for a period of 1 year. Nine (60%) of these auxiliary nursing trainees had 2–3 years and six (40%) of them had 4–6 years of experience in the nursing profession. The experiences of auxiliary nursing trainees of poverty alleviation regarding nursing and nursing profession were explored and described. Indeed, it was found that they had both negative and positive experiences and they were willing to provide quality care for their patients. The results revealed two themes and sub-themes as experiences of poverty alleviation programme trainees regarding nursing profession as presented in Table 2.

**TABLE 2:** Themes and sub-themes as experiences of poverty alleviation programme trainees regarding nursing and nursing profession.

Themes	Sub-themes
(1) Experiences of being a nurse and about nursing as a profession	1.1 Positive description of training as a nurse and the nursing profession
	1.2 Negative description of training as a nurse and the nursing profession
(2) Interpersonal relations between auxiliary nurses and the ward staff	2.1 Negative attitudes towards auxiliary nurses

#### Theme 1: Experiences of being a nurse and about nursing as a profession

Participants revealed different experiences of being nurses and about the nursing profession. Few (33.3%) participants narrated positive perspectives concerning the nursing profession – like nursing being viewed as an international profession with benefits, attractive benefits allocation to the nursing profession and availability of learning opportunities. Sub-themes that emerged under this theme were: positive description of training as a nurse and about nursing as a profession and negative description of training as a nurse and about nursing as a profession.

**Sub-theme 1.1: Positive description of training as a nurse and nursing profession:** The auxiliary nursing trainees of the poverty alleviation programme described the nursing profession as being good. They were satisfied with how they were introduced to the nursing profession. It was indicated that nursing as a profession has attractive benefits (Safadi et al. 2011). It was also interesting to note that even those who indicated that they did not like the nursing profession appreciated that they are working as nurses. The following verbatim responses were made:

‘After my training as an auxiliary nurse through poverty alleviation I knew that I can work in any hospital in South Africa and outside the country. I like nursing profession because it is recognized worldwide, a nurse can work anywhere in the world as long as you have got your certificate.’ (Participant 9, female, aged 27)

This was supported by participant 15 when saying:

‘Eh… one thing I like about nursing profession is that you don’t stay in one rank, you climb the ladder of the profession to higher ranks, and if you are studying it’s much quicker.’ (Participant 9, female, aged 27)

On the aspect of availability of learning opportunities, participants believed that challenges encountered within the nursing profession allowed them to learn more things. In support of this view, the following statements were made:

‘I have discovered that in the nursing profession you learn new things every day, even the old nurses you find them asking questions from doctors and colleagues.’ (Participant 1, female, aged 26)

In support of this quote, participant 5 said:

‘In nursing learning takes place every day, there is always something new that a nurse can learn from most cases. Some of the sisters will call me to come and assist them, that is where I will be learning some new procedures, and that makes me proud.*’* (Participant 5, male, aged 30)

This was supported by Abdel El-Halem et al. (2011) when saying ‘an independent profession by which nurses make decisions for themselves’.

**Sub-theme 1.2: Negative description of training as a nurse and the nursing profession:** Nine (60%) participants had negative experiences during their training. They reflected how their socio-economic status influenced their career choice. Participants indicated that the nursing profession was not their first priority in career choice and that they were in the nursing profession because of their situation of poverty, and as from a child-headed family. This was supported by the following responses from participant 12:

‘After passing my Grade 12 there was no money to further my studies at the University, ehh… it was tough, I wanted to study for BCom in Accounting, unfortunately my mother passed on. Nursing was not in my mind, and I still see myself as an accountant. When I further my studies I will study for BCom in Accounting.’ (Participant 12, female, aged 30)

The quote was supported by participant 6 when saying:

‘It frustrates me when we have to attend to a very ill patient being junior nurses. If I had money I will choose another career not nursing, after all it was not my intention to become a nurse. Coming to the nursing profession was my last option in the careers I had in mind. If I was given a choice or my situation allowed, I would have go for law degree. When my parents were still alive I wanted to be a lawyer and I still want to go to the University and do law.’ (Participant 6, female, aged 31)

The majority of the participants indicated that they disliked the nursing profession because of the long working hours. This is supported by the responses of participant 2:

‘Nursing profession is good, but what stress me are the long shifts and long days we are working. I can’t take care of my sibling’s problems if I am at work; I have to wait for those long days until I am off duty to get home.’ (Participant 2, female, aged 25)

Long and irregular working hours and night duty contribute to the withdrawal of nurses from the social activities of the wider community. Working on night duty was strenuous because of shortage of staff. Participant 12 said:

‘On night duty there is no support at all, you will be doing the routine alone, and you are sent up and down by the sister and they expect you to finish your work.’ (Participant 12, female, aged 30)

#### Theme 2: Interpersonal relations between auxiliary nurses and the ward staff

Participants experienced poor interpersonal relationships with their supervisors. They felt harassed, called names and were in most instances used as scapegoats for any wrong doing in the wards. They felt that registered nurses were even reluctant to work and to supervise auxiliary nursing trainees of the poverty alleviation programme. The sub-theme that emerged from this theme indicated that trainees experienced negative attitudes from the registered nurses.

**Sub-theme 2.1: Negative attitudes towards auxiliary nurses:** Participants had a feeling of not being accepted well in the wards; they were labelled and bullied by registered nurses. The findings revealed that registered nurses and staff nurses labelled auxiliary nursing trainees of poverty alleviation programme as ignorant, lazy and stubborn. It was also found that they called them names. This was confirmed by participant 8 when saying:

‘Other nurses did not accept us very well; we were called by names like “poor” kids. If anything goes wrong in the ward, you will hear them saying the poverty nurses did it, always you will try to correct mistakes done by others so that you are not called by names.’ (Participant 8, female, aged 24)

Participants further indicated that they experienced bullying behaviours from the registered and enrolled nurses. Some of the registered nurses criticise the auxiliary nursing trainees in front of patients, some show aggression towards them in front of other staff, still others gossip and direct belittling gestures at them in the wards. In support of this view, participant 14 said the following:

‘I reported a patient to a doctor during doctors’ rounds who was having a fit at the time of rounds, after the doctor has attended to the patient and left. The sister-in-charge called me and shouted at me in front of the patients saying that I am too forward; my office is the sluice room, mmm… I felt so little. Sometimes I felt that the sister-in-charge would always pick on me. She once asked me “Do you think nursing is for you?” That was so discouraging.’ (Participant 14, female, aged 25)

Experiencing of negative attitudes was further cited by participant 3 who said:

‘The sister-in-charge in male ward put it clear in front of everyone in the ward that she does not trust us nurses who are trained under poverty alleviation because we came to nursing for one reason ‘money’. I was so embarrassed and I knew it was not true.’ (Participant 3, male, aged 34)

Participants experienced bullying as normal as participant 4 supported by saying:

‘Some of the supervisors used to say “these” kids are not nurses because they are taken in the nursing profession through poverty, nursing is not their calling.’ (Participant 4, female, aged 30)

Participant 14 supported by saying:

‘One day when we entered the ward one of the sisters said to her colleagues here are “ama-poverty” (poor kids) coming, what are we going to do with them, they are not nurses these kids. We were so afraid, we were three of us in that ward; we were sent everywhere, even to the shops to buy their lunch hahaha … (laughing).’ (Participant 14, female, aged 25)

Nurses who have just entered the workplace are at great risk of being targeted for bullying as they are often younger, less experienced and somewhat insecure in their new role, and they are less aware of a unit’s cultural norms than their more seasoned colleagues (Flateau-Lux & Gravel 2014).

### Discussion of findings

Participants had positive expressions about training as nurses. The findings concurred with the study by Anurag et al. (2011) that nursing students recognised nursing as a caring profession and as an opportunity to help people gain better health. Nursing was also viewed as a noble and well-regarded career path and one that requires strength, patience and compassion (Abdel El-Halem et al. 2011). Apart from the traditional positive perception of nursing as caring, a longitudinal study that examined nursing students’ experiences of nursing considered nursing as a profession that was based on scientific knowledge, expertise and responsibility. The authors also viewed nursing as a ‘medical–technical’ activity (Lliya 2011), a career that offers employment opportunities and which promotes personal development (Mkala 2013). In a study by Stephens (2015), the values, attitudes and beliefs of students towards nursing were positive because nursing was stimulating, well-paying, respected, useful to society and was viewed as an occupation for smart people. Al Jarrah (2013) shared the same sentiments that nursing was chosen because of availability of career opportunities, jobs security, salary and interest in nursing.

In their study, Wilkes, Cowin and Johnson (2015) showed that students choose nursing because it offered work abroad and opportunities for further professional development. Improving professional practice and enhancing nurses’ clinical competence through ongoing education may increase retention and provision of quality care to patients. This statement was supported by Babiker et al. (2014) who stated that health care users benefit when the health care professional is developed.

The managers and supervisors of the institutions are responsible for developing employees, especially the neophyte nurses like the auxiliary nurses, so that they can adjust to the changing job requirements. This perspective was shared by Letlape (2012) that supervisors should continuously assess gaps in the knowledge levels of their subordinates to provide planned in-service training for corrective measures.

However, Safadi et al. (2011) in a study of 1000 American nursing students viewed nursing to be physically challenging and found that there was inadequate respect and recognition of nursing.

Contrary to the positive notions about training as nurses, participants also had negative views. The notion was also expressed by Mokoka, Oosthuizen and Ehlers (2010), in their study on retaining professional nurses in South Africa, that inflexible hours, long shifts and mandatory overtime caused disillusionment, which influenced nurses to look for other jobs. It was noted that older nurses were forced to retire earlier than they intended because of the strain of long working hours and of being overworked. Younger nurses were reported to be unhappy with shifts that impacted negatively on their families and social lives. Similarly, Banakhar (2017) discovered factors that contributed to burnout owing to overwork and extra hours beyond their normal shift hours.

Auxiliary nursing trainees in this study were not supported either by their colleagues or their supervisors in the wards. Hämmig (2017) noted that stress in a work relationship was evidenced by lack of support or help from colleagues or supervisors and poor communication between managers and employees. The auxiliary nurse trainees became powerless, humiliated and confused as they did not expect these behaviours from either their supervisors or colleagues. They felt bullied. Bullying usually involves ‘power hunger’, with the bully in a position of power compared to the victim; bullying degraded, offended and humiliated the trainees (Alswaid 2014). Stokowski (2010) said bullying made victims feel defenceless and lose their dignity at work. The researcher in this study considered that bullying in the workplace robbed the participants of decision-making power and of provision of quality care to patients. According to Treadgill (2013), some nurses viewed bullying as a normal part of the job and therefore tolerable.

This view was supported by Berry et al. (2012) that bullying is reinforced by the hierarchical organisational culture within the hospital which limits reporting of bullying behaviours.

Some of the auxiliary nurse trainees viewed bullying from their seniors as being strict, and they accepted it as a normal phenomenon in the working place. Auxiliary nurses appeared to be incorrectly mentored and utilised by the staff in the wards, as some were delegated to a number of non-nursing duties as well as duties above or outside their scope of practice.

### Limitations of the study

The literature review for this area was limited because the topic has not yet been researched and this influenced the literature control. The researchers used open coding method for data analysis, as they were not able to use the computer software to analyse qualitative data. Only four hospitals were involved in Vhembe District. Findings will not be generalised to other hospitals in Vhembe and in the Limpopo Province.

### Recommendations

It is recommended that professional nurses in the wards be aware of the experiences, so that they can create a conducive environment in which the training and education of auxiliary nursing trainees take place in a professional manner. The auxiliary nurses need constant and direct supervision because when qualified, they will be responsible for direct and/or indirect nursing care of a patient or a group of patients (but still under supervision). Regular in-service-education sessions should be given about the scope of practice not only to nursing auxiliaries themselves but also to professional and enrolled nurses who act as supervisors to nursing auxiliaries.

## Conclusions

This study revealed that auxiliary nursing trainees of the poverty alleviation programme felt that nursing profession was not their career of choice; they became nurses because of their situations. They felt unaccepted by some of the staff members because they were taken from home to train as nurses, while other participants always wanted to become nurses and they used the opportunity to realise their dreams. Professional nurses must have the responsibility to prepare the auxiliary nurses, irrespective of their socio-economic status, to function as competent professionals who have the knowledge and the ability to apply skills according to the scope of practice.

## References

[CIT0001] Abdel El-Halem G.E., El Hawashy Z.I., Gamal El-Dein A.A. & Taha E.E., 2011, ‘Undergraduate male nursing students’ perception about the image of the nursing profession’, *Journal of American Science* 7(3), 614–623.

[CIT0002] Al Jarrah I.A.T., 2013, ‘Associate nursing students’ perceptions toward nursing profession in Jordan’, *European Scientific Journal* 9(6), 147–166.

[CIT0003] Alswaid E., 2014, ‘Workplace bullying among nurses in Saudi Arabia: An exploratory qualitative study’, Unpublished theses presented in partial fulfilment of the requirements for the degree of Master of Management at Massey University in Saudi Arabia.

[CIT0004] ANC, 2003, *A year of the struggle against Poverty*, Umanyano Publication, Johannesburg.

[CIT0005] Anurag B., Patidar J.K., Suresh K.S. & Neeraj S., 2011, ‘Future nurses’ perception towards profession and carrier plans: A cross sectional survey in state Punjab’, *Nursing and Midwifery Research Journal* 7(4), 176.

[CIT0006] Babiker A., El Hussini M., Al Nemri A., Al Frayh A., Al Juryyan N., O Faki M. et al., 2014, ‘Health care professional development: Working as a team to improve patient care’, *Sudanese Journal of Paediatrics* 14(2), 9–16. 27493399 PMC4949805

[CIT0007] Banakhar M., 2017, ‘The impact of 12-hour shifts on nurses’ health, wellbeing, and job satisfaction: A systematic review’, *Journal of Nursing Education and Practice* 7(11), viewed 24 November 2016, from http://jnep.sciedupress.com.

[CIT0008] Berry P.A., Gillespie G.L., Gates D. & Schafer J., 2012, ‘Novice nurse productivity following workplace bullying’, *Journal of Nursing Scholarship* 44, 80–87. 10.1111/j.1547-5069.2011.01436.x 22339938 PMC12126179

[CIT0009] Creswell J.W., 2014, *Research design qualitative, quantitative and mixed methods approaches*, 4th edn., Sage, Thousand Oaks, CA.

[CIT0010] De Vos A.S., Strydom H., Fouche C.B. & Delport C.S.L., 2014, *Research at grass roots: For the social sciences and human service professions*, 7th edn., Pretoria, Van Schaik.

[CIT0011] Department of Health and Social Development, 2003, *Guidelines for auxiliary nurse training, poverty alleviation programme*, Nursing Education, Limpopo Government, Republic of South Africa, Pretoria.

[CIT0012] Final Limpopo DHSD 2008/09-11 Annual Performance Plan – Vote 7, 2011, viewed 01 April 2018, from http://www.dhsd.limpopo.gov.za/docs/reports/final.

[CIT0013] Flateau-Lux L.R. & Gravel T., 2014, ‘Put a stop to bullying new nurse’, *Home Healthcare Now* 32(4), 225–119. 10.1097/NHH.0000000000000045 24685753

[CIT0014] Guba E.G. & Lincoln Y.S., 1985, *Naturalistic enquiry*, Sage, Beverly Hills, CA.

[CIT0015] Hämmig O., 2017, ‘Health and well-being at work: The key role of supervisor support’, *Science of Medicine – Population Health* 3, 393–402. 10.1016/j.ssmph.2017.04.002 PMC5769068 29349232

[CIT0016] Kgadima N.P., 2009, ‘The understanding of poverty-alleviation project participants’, Unpublished Masters Dissertation, in Social Science (Mental Health) University of South Africa.

[CIT0017] Letlape H.R., 2012, *The exploration of in-service training needs of psychiatric nurses*, viewed from http://dspace.nwu.ac.za/bitstream/handle/10394/8451/Letlape_HR.pdf.

[CIT0018] Letshokgohla M.E., 2009, ‘Evaluation of the MEC’s poverty alleviation programme in the Waterberg District of the Limpopo Province, Unpublished Masters Dissertation, in Public Health Sciences, University of Witwatersrand.

[CIT0019] Lliya W., 2011, *Assessment of nurses’ perception towards nursing profession in public hospitals under Addis Ababa Health Bureau, Ethiopia*, viewed 11 October 2016, from http://etd.aau.edu.et/dspace/handle/123456789/2446.

[CIT0020] Mayombe C., 2014, ‘Assessment of non-formal adult education and training centres’ enabling environments for employment and poverty reduction in KwaZulu-Natal, South Africa’, PhD thesis, University of Pretoria, Pretoria.

[CIT0021] Mkala B., 2013, *Nursing as a career: First year students’ perception of and the reason for their choice of nursing as a career*, Degree Programme in Nursing Social Services, Health and Sport, Unpublished thesis, JAMK University of Applied Sciences.

[CIT0022] Mokoka E., Oosthuizen M.J. & Ehlers V.J., 2010, ‘Retaining professional nurses in South Africa: Nurse Managers’ perspective’, *Health South Africa, Gesondheid* 15(1), 1–30.

[CIT0023] Sachs J.D. & McArthur J.W., 2005, ‘The Millennium project: A plan for meeting the Millennium development goals’, *The Lancet* 395. 10.1016/S0140-6736(05)17791-5 15664232

[CIT0024] Safadi R.R., Saleh M.Y.N., Nassar O.S., Amre H.M. & Froelicher E.S., 2011, ‘Nursing students’ perceptions of nursing: A descriptive study of four cohorts’, *International Nursing Review* 58(4), 420–427. 10.1111/j.1466-7657.2011.00897.x 22092319

[CIT0025] Sekoati S., 2008, *Speech delivered by MEC for health and social development Limpopo Province*, viewed 26 September 2016, from http://www.info.gov.za/aboutSA/health.htm.

[CIT0026] South African Nursing Council, 2005, *Philosophy and policy of the South African Nursing Council with regard to professional nursing education and training*, SANC, Pretoria.

[CIT0027] Stephens M., 2015, ‘Changing student nurses values, attitudes and behaviours: A meta-ethnography of enrichment activities’, *Journal of Nursing Care* 5, 320 10.4172/2167-1168.1000320

[CIT0028] Stokowski L.A., 2010, ‘A matter of respect and dignity: Bullying in the nursing profession’, *Journal of Nursing Academic* 24(3), 12–14.

[CIT0029] Treadgill C.R., 2013, *Perceptions of workplace bullying among practicing registered nurses*, viewed 28 February 2017, from http://aquila.usm.edu/dissertation/747.

[CIT0030] Wilkes L., Cowin L. & Johnson M., 2015, ‘The reasons students choose to undertake a nursing degree’, *Collegian* 22, 259–265. 10.1016/j.colegn.2014.01.003 26552196

